# Improving Prediction Algorithms for Cardiometabolic Risk in Children and Adolescents

**DOI:** 10.1155/2013/684782

**Published:** 2013-06-19

**Authors:** Ulla Sovio, Aine Skow, Catherine Falconer, Min Hae Park, Russell M. Viner, Sanjay Kinra

**Affiliations:** ^1^Department of Non-Communicable Disease Epidemiology, London School of Hygiene and Tropical Medicine, Keppel Street, London WC1E 7HT, UK; ^2^General and Adolescent Paediatrics Unit, Institute of Child Health, University College London, 30 Guilford Street, London WC1N 1EH, UK

## Abstract

Clustering of abnormal metabolic traits, the Metabolic Syndrome (MetS), has been associated with an increased cardiovascular disease (CVD) risk. Several algorithms including the MetS and other risk factors exist for adults to predict the risk of CVD. We discuss the use of MetS scores and algorithms in an attempt to predict later cardiometabolic risk in children and adolescents and offer suggestions for developing clinically useful algorithms in this population. There is little consensus in how to define the MetS or to predict future CVD risk using the MetS and other risk factors in children and adolescents. The MetS scores and prediction algorithms we identified had usually not been tested against a clinical outcome, such as CVD, and they had not been validated in other populations. This makes comparisons of algorithms impossible. We suggest a simple two-step approach for predicting the risk of adult cardiometabolic disease in overweight children. It may have advantages in terms of cost-effectiveness since it uses simple measurements in the first step and more complex, costly measurements in the second step. It also takes advantage of the continuous distributions of the metabolic features. We suggest piloting and validating any new algorithms.

## 1. Introduction

Obesity is associated with a number of diseases, such as type 2 diabetes (T2D), cardiovascular disease (CVD), cancers, asthma, osteoarthritis, chronic back pain, and sleep apnoea [1]. As childhood obesity prevalence has risen markedly in recent decades [2], indicators of early development of some of these diseases are increasingly being seen in childhood and adolescence. For example, the lowest estimated prevalences of impaired glucose tolerance, hypertension, and raised total cholesterol in obese 5–18-year-old children in the European Union in 2006 were 8.4%, 21.8%, and 22.1%, respectively [3]. Obesity and its associated health risk factors track strongly from childhood into adulthood [4]. Therefore, identification of children at an increased risk of developing obesity-related diseases is critical for early prevention.

Various algorithms have been developed for adults to provide individual predictions of risk of obesity-related diseases, particularly of cardiometabolic risk leading to CVD [5–15]. These include the widely used Framingham Risk Score [6, 7], which uses information on age, sex, blood pressure, total cholesterol (TC), high density lipoprotein cholesterol (HDL-C), diabetes, and current smoking behaviour to give an estimate of 10-year CVD risk in adults aged ≥20 years. 

Attempts have also been made to develop similar algorithms to predict cardiometabolic risk in children [16–27], and most of them focus on identifying the Metabolic Syndrome (MetS, the clustering of abnormal metabolic traits associated with CVD risk) [28, 29]. However, these algorithms are not widely used. There is little consensus on the criteria for defining the MetS in children and adolescents, and most algorithms have not been validated. The existing childhood MetS definitions have been derived from adult definitions assuming that the conditions are related over the life course, while the utility and predictive value of MetS in children have not yet been fully established [30]. In this review, we examine the benefits and limitations of MetS scores and algorithms that have been developed to predict later cardiometabolic risk in children and adolescents and offer suggestions for developing clinically useful algorithms in this population. Such algorithms could aid primary care professionals in the identification of children at high risk of obesity-related diseases and would be an important tool in childhood obesity management.

## 2. Using Metabolic Syndrome (MetS) Scores to Predict Cardiometabolic Risk

The MetS in adults is characterised by obesity (often assessed by large waist circumference (WC)), high triglycerides (TG), low HDL-C, high blood pressure, and high glucose levels [25]. The prevalence of the MetS increases with age. In children and adolescents, the thresholds for the individual variables to define these “high” and “low” levels depend on the age of the population and the MetS definition applied.

Notably, because there is no consensus about the definition of MetS in children, it can be difficult to make consistent and accurate diagnoses [27]. Due to the lack of universal MetS definition and for the sake of retaining statistical power, construction of continuous MetS scores (cMetS) has gained popularity [31]. Many algorithms which predict cardiometabolic risk in children are based on these MetS scores, with children who are classified as having MetS or children with a high value of cMetS being flagged as having an increased future cardiometabolic risk. Studies that have used either continuous [16] or binary [24, 26, 27] MetS definitions/scores to assess or predict future cardiometabolic risk have been reviewed previously. These studies do not always explicitly state the future disease that they are trying to predict (e.g., CVD); instead, terms such as cardiovascular or cardiometabolic risk are widely used. The association between MetS characteristics in childhood and increased adult risk of CVD may largely be due to tracking of obesity and other MetS characteristics from childhood into adulthood [4]. 

One recent example of a MetS definition is from the International Diabetes Federation (IDF), which modified a definition originally developed for adults. The IDF has age-specific definitions of MetS (6–<10, 10–<16, and ≥16 years) [25, 26] which is an improvement over earlier definitions that did not include age stratification [26, 27]. Except for the youngest age group (where MetS is not defined, but further measurements for obese children with a family history of CVD or related conditions are recommended), the IDF definition produces a binary outcome identifying children as having MetS if the child has high WC and exceeds threshold levels for at least two of the following four risk factors: high TG, low HDL-C, high SBP/DBP, and high glucose. In that sense, it is similar to earlier MetS definitions in paediatrics [16, 24, 26, 27]. However, the IDF definition has been regarded as the most appropriate binary MetS definition in children since it applies different criteria for each age group, acknowledging that blood pressure, lipid levels, and anthropometric variables change with age and pubertal development [27]. If the IDF definition in children becomes widely accepted, it could be used to aid in the identification of children at an elevated cardiometabolic risk. Alternatively, to maximise the use of information and statistical power, the underlying continuous risk factors that compose the IDF definition from age 10 years could be turned into a cMetS. The threshold indicating a high value of this score could then be defined for each age group or ever for each age (in years) to increase accuracy.

## 3. Algorithms for Cardiometabolic Risk Prediction Which Include Items in addition to MetS Components

Some algorithms for predicting cardiometabolic risk in children include additional characteristics to metabolic traits that are established risk factors of CVD. Examples include models which additionally use ethnicity, family history of disease, fitness level, or smoking status to compute a final risk score (Table 1) [18–23, 32–36]. In these algorithms, however, the choice of additional non-MetS components is rarely justified and in many cases appears to be based solely on availability of data or characteristics identified in studies restricted to adult populations. 

Diagnostic test results for most of the algorithms developed in children have not been published, in most cases due to the lack of current or subsequent clinical outcomes (such as CVD) against which they could have been tested. We did, however, find two exceptions. One study—which predicted the probability of developing atherosclerotic lesions in the coronary artery and abdominal aorta based on age, sex, smoking, BMI, hypertension, hyperglycemia, HDL cholesterol, and non-HDL cholesterol—reported an area under the receiver-operating curve (AUC) of 0.78 for the risk of coronary artery lesions and of 0.84 for abdominal aorta lesions [21]. Another study tested a score based on the refined American Diabetes Association (ADA) criteria for glucose intolerance in a clinical high-risk population from Birmingham; this study reported sensitivity of 100%, specificity of 57%, and positive predictive value (PPV) of 36% [36]. 

We did not identify any validation studies of these risk algorithms in populations other than those in which they were first developed or applied. Without these two important steps of testing and validation, it is impossible to compare the performance of different algorithms to one other. Testing and validation are needed to develop consensus on what is the best method for predicting cardiometabolic risk in children. 

## 4. Improving Cardiometabolic Risk Algorithm for Children

Using a summary score to predict cardiometabolic risk in children, like all the MetS scores reviewed here, is appealing because it reduces different metabolic dimensions into a single variable. However, certain assumptions have to be made when this approach is used [31]. Each item included in the score is assumed to be equally important (unless weighting is used) and exchangeable with any other item. For example, 1 SD increase in blood pressure is assumed to be equivalent with 1 SD increase in TG or 1 SD decrease in HDL-C. These assumptions are unlikely to reflect reality, and they may be difficult to validate in real datasets. 

Instead of using a binary MetS outcome, which is a common practice, a continuous MetS score (cMetS) may be preferable. This can be a sum of individual rankings, sum or mean of z-scores, principal components, or sum of standardised residuals [16]. This approach overcomes problems such as misclassification and low statistical power often related to binary scores. However, if the resulting cMetS is subsequently grouped using cutoffs, like the age-specific IDF MetS definition [26], misclassification may still occur. Researchers have been urged to create and validate population-specific cMetS for children [31]. Although this approach is justifiable, it complicates comparisons between populations.

There are also some general issues concerning screening tools that need to be taken into account when they are applied in clinical practice. If the sensitivity of the tool is not adequate, a considerable proportion of children at risk will not be detected. On the other hand, if specificity of the tool is limited, some children who are not at an increased risk will be captured and may have to go through unnecessary further testing which may cause anxiety to the children and their families. A test with reasonably good sensitivity and specificity may be useful for early identification of children with increased future risk, which in turn may create opportunities for early intervention. For example, in the study which predicted the probability of developing atherosclerotic lesions [21], AUC based on sensitivity and specificity was fairly good, but the results of this study were not replicated. 

The applicability of an algorithm should always be piloted in the target population before implementing it, since an algorithm developed in one population may not be useful in another population without modification. For example, some algorithms have been developed in selecting clinical populations [36] or in ethnic groups of a higher risk. This is especially a concern when cMetS is used and when cut-off points for an increased risk are defined without a clinical basis [31]. 

We suggest development of an algorithm which fully utilises the continuous distributions of metabolic features (e.g., by calculating z-scores). For example, a two-step approach may have advantages in terms of cost-effectiveness. The first step would target a wider range of overweight/obese children, perhaps through primary care, and those who were flagged as overweight (using age, sex, and potentially ethnicity standardised BMI z-score) could be assessed for other easily measurable factors predictive of later cardiometabolic disease, for example, blood pressure. The small group of children that exceed a stringent threshold for a standardised summary score for estimated risk could then be taken forward to the next step. In this next step, more complex, costly, or time-consuming clinical measurements (e.g., lipids, insulin, and glucose) could be taken from referred children and an updated standardised risk score could be calculated. Children with a “high risk” score should be offered specialised weight management interventions if these are available or be monitored regularly and treated when necessary. This approach combines the benefits of the use of continuous metabolic features in the first step while retaining usability through the application of cutoffs for flagging children who are predicted to be at the highest risk.

The suggested algorithm could be based on a fitted prediction model in a large, longitudinal, population-based dataset that covers anthropometric and metabolic data and other relevant risk factors from childhood and adolescence as well as clinical outcomes (T2D, hypertension, and CVD) from adulthood. The algorithm should be validated and refined in other datasets. Once validated, it should be an improvement over existing algorithms because it uses complete information from the continuous distributions of the metabolic risk factors.

Our work using a large UK cohort [37] suggested that age, sex, and ethnicity standardised BMI z-score was the best predictor of having cardiovascular risk factors (elevated glucose, LDL-C, and/or SBP/DBP) present in childhood (submitted manuscript). Based on this work, we created a simple online tool that gives primary care providers guidance on how to treat and appropriately refer children who are overweight. This tool was piloted among future user groups in the UK. Validation against clinical cardiometabolic outcomes in adulthood was not possible using the same cohort due to its young age, but we hope to validate our models in larger, more diverse datasets in the future.

## 5. Conclusions 

Several different MetS scores and algorithms which predict adult cardiometabolic risk in children have been developed, but diagnostic test results against a clinical outcome, such as CVD, have not been published for most of them, and they have not been validated in other populations. We suggest a simple two-step approach for predicting risk of adult cardiometabolic disease in overweight children and piloting and validating any new algorithms.

## Figures and Tables

**Table 1:** Cardiometabolic risk scores developed for children and adolescents.

	Andersen and Haraldsdottir 1993 [32]	ADA 2000 [22, 36]	Rodríguez-Morán et al. 2004 [23]	McMahan et al. 2005 [21]	Andersen et al. 2006 [33]	Reed et al. 2007 [18]	Brambilla et al. 2007 [19] Bueno et al. 2007 [20]	Andersen et al. 2010 [35]
*N*	203	66	965	2,575	1,732	242	153	210
Age range (years)	15–19	5–17	10–18	15–34	9–15	9–11	9–13	9-10
Sex (% male)	43	29	N/A	N/A	47	50	52	54

Nationality or ethnicity	Danish	South Asian, British, and African Caribbean	Mexican		Danish, Estonian, and Portuguese	Canadian	Spanish	Danish

Stratification, adjustment, or standardisation	Sex	Age and sex (for BMI)	Age and sex (for BMI, BP, and TG)		Age, sex, and country	Age, sex	Sex (for obesity)	Sex

Exclusions							Nonobese	

Score name	Total risk score	T2D criteria	REGODCI	PDAY	Composite score	Healthy Heart Score	MIRACLE	Composite risk factor score

Risk factor included [Y = yes]								

Age				Y				
Sex				Y				
BMI		Y	Y	Y		Y	Y	
WC*							*Y *	
Skinfolds	Y				Y			Y
*SB* P*	*Y *		*Y *		*Y *	*Y *	*Y *	*Y *
*DB* P*	*Y *		*Y *			*Y *	*Y *	
*Hy* *pe* *rt* *en* *si* on*		*Y *		*Y *				
TC	Y							
*HD* *L*-C*	*Y *		*Y *	*Y *				
Non-HDL-C				Y				
TC:HDL-C					Y			Y
TG*	*Y *		*Y *		*Y *			*Y *
*Gl* *uc* *os* e*			*Y *					
Dyslipidemia		Y						
Hyperglycemia				Y			Y	
Insulin resistance					Y			Y
T2D							Y	
PCOS		Y						
Smoking	Y			Y				
Fitness					Y	Y		Y
PA						Y		
Family history of CVD/T2D/Hypertension/obesity		Y	Y				Y	
SGA							Y	
Birth weight			Y					
Ethnicity		Y					Y	
Acanthosis nigricans		Y					Y	

*MetS components are given in italics.

## Acronyms

CVD: Cardiovascular disease
CHD: Coronary heart disease
MetS: Metabolic Syndrome
cMetS: Continuous metabolic syndrome score
T2D: Type 2 diabetes
BMI: Body mass index
WC: Waist circumference
SBP: Systolic blood pressure
DBP: Systolic blood pressure
MAP: Mean arterial pressure
TG: Triglyceride
TC: Total cholesterol
HDL-C: High density lipoprotein cholesterol
TC : HDL-C: TC/HDL-C ratio
LDL-C: Low density lipoprotein cholesterol
IR: Insulin resistance
HOMA: Homeostasis model assessment
IDF: International Diabetes Federation
IGT: Impaired glucose tolerance
OGTT: Oral glucose tolerance test
PCOS: Polycystic ovarian syndrome
ROC: Receiver operating characteristic
PA: Physical activity
SGA: Smallness for gestational age
PPV: Positive predictive value.
